# Direct Immobilization of Engineered Nanobodies on Gold Sensors

**DOI:** 10.1021/acsami.1c02280

**Published:** 2021-04-12

**Authors:** Bárbara Simões, Wanda J. Guedens, Charlie Keene, Karina Kubiak-Ossowska, Paul Mulheran, Anna M. Kotowska, David J. Scurr, Morgan R Alexander, Alexis Broisat, Steven Johnson, Serge Muyldermans, Nick Devoogdt, Peter Adriaensens, Paula M. Mendes

**Affiliations:** † School of Chemical Engineering, University of Birmingham, Edgbaston, Birmingham B15 2TT, United Kingdom; ‡ Institute for Materials Research (IMO), Hasselt University, BE-3590 Diepenbeek, Belgium; § Department of Physics, University of Strathclyde, Glasgow G1 1XQ, United Kingdom; ∥ School of Pharmacy, University of Nottingham, Nottingham NG7 2RD, United Kingdom; ⊥ Department of Electronic Engineering, University of York, York YO19 5DD, United Kingdom; # Laboratory of Bioclinical Radiopharmaceutics, Université Grenoble Alpes, Inserm, CHU Grenoble Alpes, LRB, 38000 Grenoble, France; ∇ Cellular and Molecular Immunology laboratory, Vrije Universiteit Brussel (VUB), BE-1050 Brussels, Belgium; ○ In vivo Cellular and Molecular Imaging laboratory, Vrije Universiteit Brussel (VUB), BE-1090 Brussels, Belgium; ◆ Department of Chemical & Process Engineering, University of Strathclyde, Glasgow G1 1XQ, United Kingdom

**Keywords:** nanobody, single-domain antibody, surface plasmon resonance, sensor, molecular dynamic simulations

## Abstract

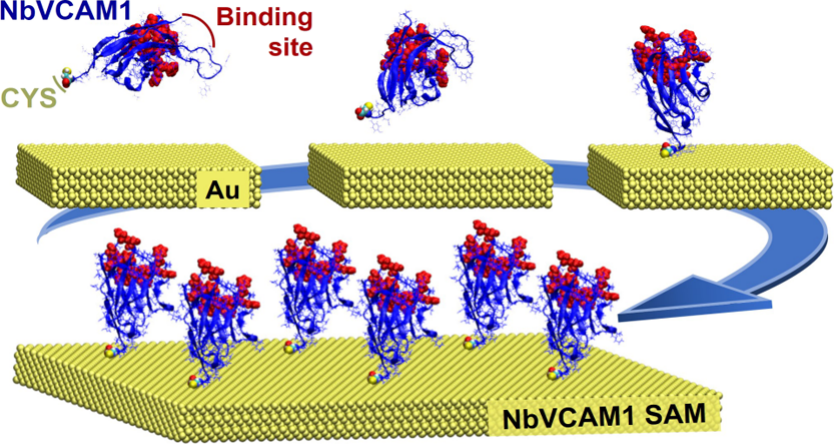

Single-domain antibodies, known as nanobodies, have great potential as biorecognition elements for sensors because of their small size, affinity, specificity, and robustness. However, facile and efficient methods of nanobody immobilization are sought that retain their maximum functionality. Herein, we describe the direct immobilization of nanobodies on gold sensors by exploiting a modified cysteine strategically positioned at the C-terminal end of the nanobody. The experimental data based on secondary ion mass spectrometry, circular dichroism, and surface plasmon resonance, taken together with a detailed computational work (molecular dynamics simulations), support the formation of stable and well-oriented nanobody monolayers. Furthermore, the nanobody structure and activity is preserved, wherein the nanobody is immobilized at a high density (approximately 1 nanobody per 13 nm^2^). The strategy for the spontaneous nanobody self-assembly is simple and effective and possesses exceptional potential to be used in numerous sensing platforms, ranging from clinical diagnosis to environmental monitoring.

## Introduction

Single-domain antibodies, generally referred to as nanobodies, are emerging as robust and versatile affinity reagents for research, diagnostics, and therapeutics.^1,2^ They are an attractive alternative to antibodies because they offer a similar high affinity and high selectivity for a broad range of analytes (small organic molecules, proteins, cell epitopes), but they are smaller in size (∼15 kDa). This latter characteristic confers them with increased solubility and stability, easier production, and low steric hindrance to reach targets.^3,4^ This unique set of properties makes nanobodies ideal building blocks for a wide range of sensing devices and assays for use in medical, biotechnology, environmental, food, and even military settings.

Despite great advances in the nanobody technology, few approaches have been reported for the immobilization of nanobodies on sensing platforms.^5−7^ Physical adsorption has been investigated for nanobody immobilization on gold nanoparticles which are used as immunoassay detection labels.^6^ While stable nanobody–gold nanoparticle conjugates can be generated,^8^ this requires careful consideration of the influence of the nanobody isoelectric point, pH, and ionic strength of the solution. Instead of relying on direct immobilization on a sensor surface, Adriaensens and co-workers^5^ established a two-step protocol in which the sensor surface was initially functionalized with an azide-terminated monolayer and then exposed to an engineered nanobody carrying a C-terminal alkyne function. Taking advantage of the copper(I)-catalyzed cycloaddition reaction (“click” chemistry), the formation of a stable and well-oriented nanobody monolayer was achieved. In a recent example, nanobodies have been tagged with histidines, which served to couple the nanobody to cobalt-nitrilotriacetic acid metal-chelate beads.^7^

In spite of these and other efforts in the literature,^9−11^ efficient, alternative immobilization methods are still needed to meet the requirements of a wide range of sensing applications. In this context, gold surfaces are widely employed as interfaces in various biochemical and chemical sensors because of their high electrical conductivity, unique optical properties, biocompatibility, and chemical stability.^12,13^ The mechanisms of these sensors are based on various detection methods, including electrochemical (impedance spectroscopy^14^ and cyclic voltammetry),^15^ piezoelectric (surface acoustic wave (SAW)^16^ and quartz crystal microbalance (QCM)^17^), and optical (e.g., surface plasmon resonance (SPR),^18^ localized surface plasmon resonance (LSPR),^19^ and surface-enhanced Raman spectroscopy (SERS)^20^) detection methods. The prevailing involvement of gold surfaces in a diversity of sensing technologies highlights the necessity for strategies that not only promote fast and robust immobilization but also promote high-efficiency target binding.

**Figure 1:**
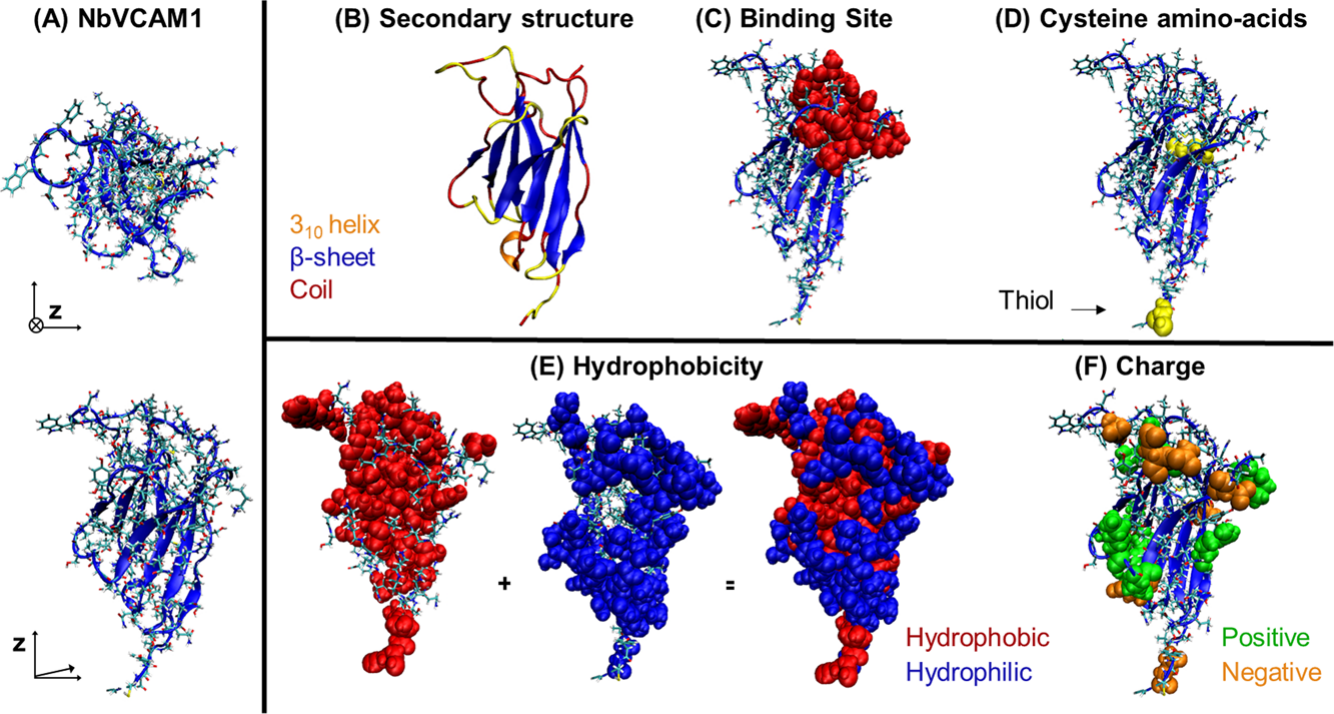
NbVCAM1 nanobody (14.5 kDa) visual molecular dynamics (VMD) images, shown by a new cartoon merged with bond representations: (A) NbVCAM1 top and side views, with dimensions 3.1 nm × 4.0 nm × 5.3 nm; (B) secondary structure with colors: 3_10_ helix (orange), β-sheet (blue), turn (yellow), and coil (red); (C) amino acids forming the antigen binding site (van der Waals representation (VDW), red) located at the N-terminus side of the domain; (D) cysteines (VDW, yellow) that form a disulfide bridge at the core and the one located at the C-terminus; (E) hydrophobic (VDW, red) and hydrophilic (VDW, blue) amino acids; (F) negative (VDW, orange) and positive (VDW, green) amino acids. The NbVCAM1 has a net charge of +2e at pH 7.0.

With this proviso in mind, in this work, we investigated the ability of an engineered nanobody comprising a modified cysteine to readily generate stable, well-oriented, and packed nanobody monolayers on gold surfaces. The expressed protein ligation (EPL) technique was used to incorporate alkyne-modified cysteine at the C-terminal of the model nanobody NbVCAM1, which targets the vascular cell adhesion molecule-1 (VCAM1).^21^ The modified cysteine group, which binds to gold via the thiol group, is located at the opposite end of the binding pocket (Figure 1). While the native nanobody contains two other cysteines and four methionines, these moieties are not expected to interact with the gold surface. The two native cysteine residues are located in the interior core of the nanobody, forming the typical disulfide bridge responsible for structural stability,^22^ which makes the moieties unlikely to interact with gold. Additionally, previous studies have shown that methionines poorly interact with gold.^23−25^

In order to obtain a detailed insight into the interface chemistry, structural stability, orientation, and activity of the immobilized nanobodies, a suite of complementary surface analysis techniques was employed, including contact angle, ellipsometry, time-of-flight secondary ion mass spectrometry (ToF-SIMS), three dimensional (3D) Orbitrap secondary ion mass spectroscopy (3D OrbiSIMS), circular dichroism (CD), and SPR. The molecular interactions occurring at the gold-nanobody interface and the stable conformation of the immobilized nanobodies are further validated using molecular dynamic (MD) simulations.

## Results and Discussion

NbVCAM1 monolayers were formed by immersing freshly cleaned gold substrates in a solution of 1 μM NbVCAM1 in phosphate buffer saline (PBS) for 24 h, which provides the time for the formation of a gold-thiolate bond between the gold surface and the NbVCAM1 nanobody.^26^ Contact angle data show the formation of a hydrophilic surface, with the NbVCAM1 monolayers exhibiting the advancing and receding contact angles of 62.6 ± 2.3 and 26.0 ± 6.5^o^, respectively. These values are comparable to those obtained for protein monolayers,^27^ with the large contact hysteresis (36.6°), indicating the presence of a heterogeneous surface due likely to the exposure at the interface of hydrophobic and hydrophilic amino acids from the nanobody and/or nanobody packing arrangement. The ellipsometric thickness observed for the NbVCAM1 monolayer is 1.99 ± 0.09 nm, which is less than the theoretical molecular length of the nanobody from the C-terminally added cysteine–alkyne linker to the N-terminal, that is, 5.3 nm (Figure 1). This discrepancy, between the molecular length and the self-assembly monolayer (SAM) thickness, can be explained by the presence of air voids between the nanobodies and within the nanobodies themselves.^28,29^

**Figure 2:**
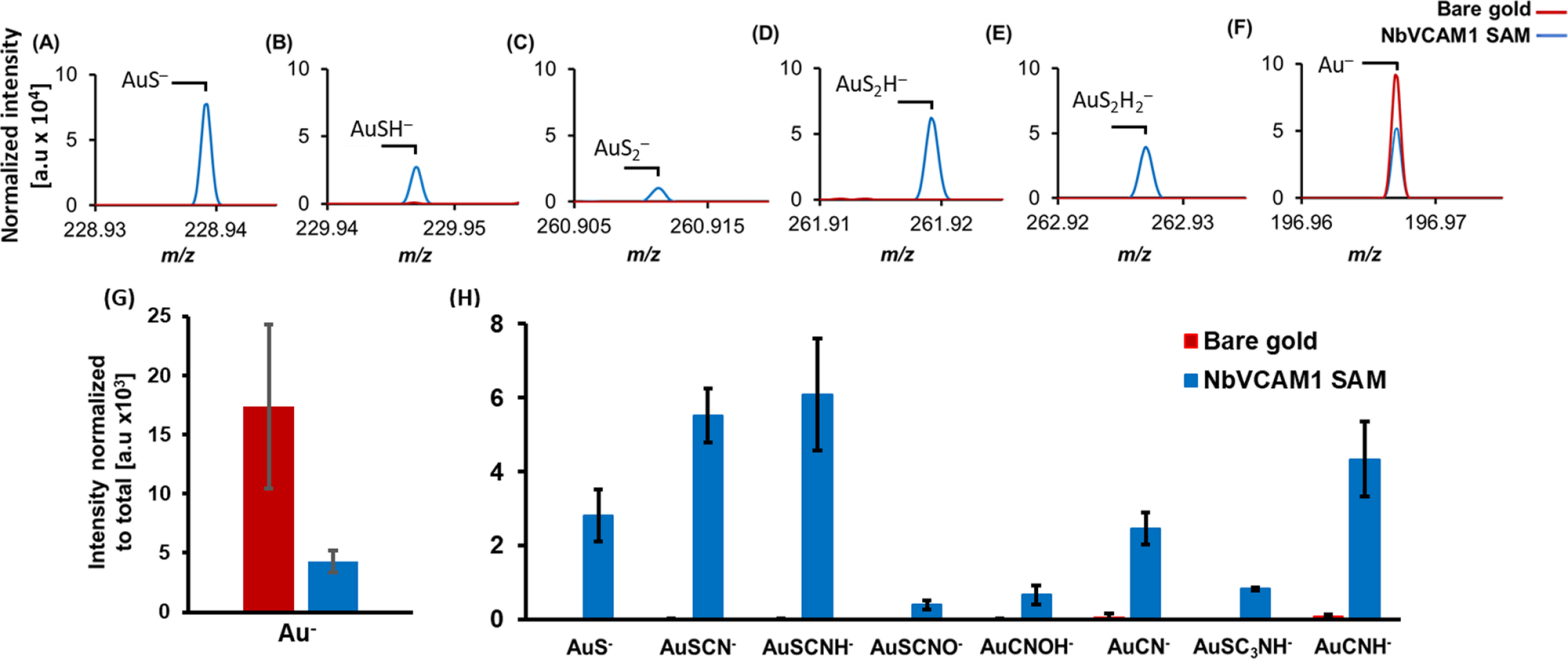
Overlay of 3D OrbiSIMS spectra (20 keV Ar_3000_^+^ as a primary ion beam) for the peak intensities of AuS^–^ and related ions (A-E) and the substrate ion Au^–^ (F) on the control bare gold (red) and NbVCAM1 SAM (blue). Intensity was normalized to the total ion counts. “a.u” refers to arbitrary units. Comparison of (G) gold and (H) gold–sulfur and gold–nitrogen containing species ion peak intensities in the gold reference samples (red) and NbVCAM1 SAM samples (blue). Average and standard deviation for four 3D OrbiSIMS measurements. Intensity was normalized to the total ion counts.

Following these initial results providing the evidence of NbVCAM1 monolayer formation, 3D OrbiSIMS and ToF-SIMS were used to investigate whether or not the gold-thiolate bond was formed upon adsorption. 3D OrbiSIMS and ToF-SIMS survey spectra (Figure S1) along with the high-resolution spectra (Figure 2) are shown for the NbVCAM1 SAM and control bare gold. The mass resolving power of the 3D OrbiSIMS allows the assignment of secondary ion peaks associated with the proposed Au-S bond (shown in Figure 2), which could not be confidently distinguished in the ToF-SIMS spectra (Figure S2). The negative polarity 3D OrbiSIMS spectra of the NbVCAM1 monolayer on gold are illustrated in Figure 2, together with clean gold as a control. Secondary ions associated with the AuS^–^ ion and related fragments (AuSH^–^, AuS_2_^–^, AuS_2_H^–^, and AuS_2_H_2_^–^) can be observed clearly for the NbVCAM1 monolayer, but they are absent in the clean gold control. Additionally, the NbVCAM1 monolayer attenuated the intensity of Au^–^ ion fragments, which were less accessible to be ionized because of the presence of NbVCAM1 (Figure 2F). In addition to providing further evidence supporting the formation of the NbVCAM1 monolayer on gold, these results also confirm the formation of a thiolate bond between the NbVCAM1 and the gold surface, wherein adventitious sulfur is excluded as a possible source of AuS^–^ ions.

**Figure 3:**
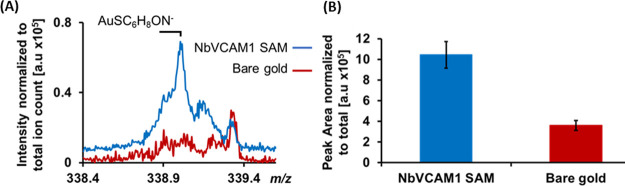
(A) ToF-SIMS spectra and (B) respective normalized peak intensity areas showing the presence of the AuSC_6_H_8_ON^–^ ion fragment on the NbVCAM1 SAM but its absence on the clean gold control surface. Average and standard deviation for three measurements over two samples. ‘a.u’ refers to the arbitrary units.

Along with the fragments associated with the Au-thiolate bond, the presence of ions containing Au and nitrogen is also observed (Figure 3A). They are present at a much higher intensity than those on clean gold surfaces, indicating the interactions between the amino-acid residues in the NbVCAM1 and the gold surface. These interactions can arise from nitrogen atoms located in positions close to the cysteine–alkyne linker because there is a possibility of the nanobody making more than one point of contact with the gold surface. However, we cannot exclude the possibility that some nanobodies might be randomly oriented on the surface. The 3D OrbiSIMS results are further supported by the ToF-SIMS analysis (TOF IV instrument with 25 keV Bi_3_^+^ primary ion beam), wherein a peak for the ion fragment AuSC_6_H_8_ON^–^ can be distinguished from the clean gold control surface (Figure 3B). This distinctive fragment belongs to the alkyne-modified cysteine, thus supporting the formation of a thiolate bond between the thiol group in the modified NbVCAM1’s cysteine and the gold surface.

**Figure 4:**
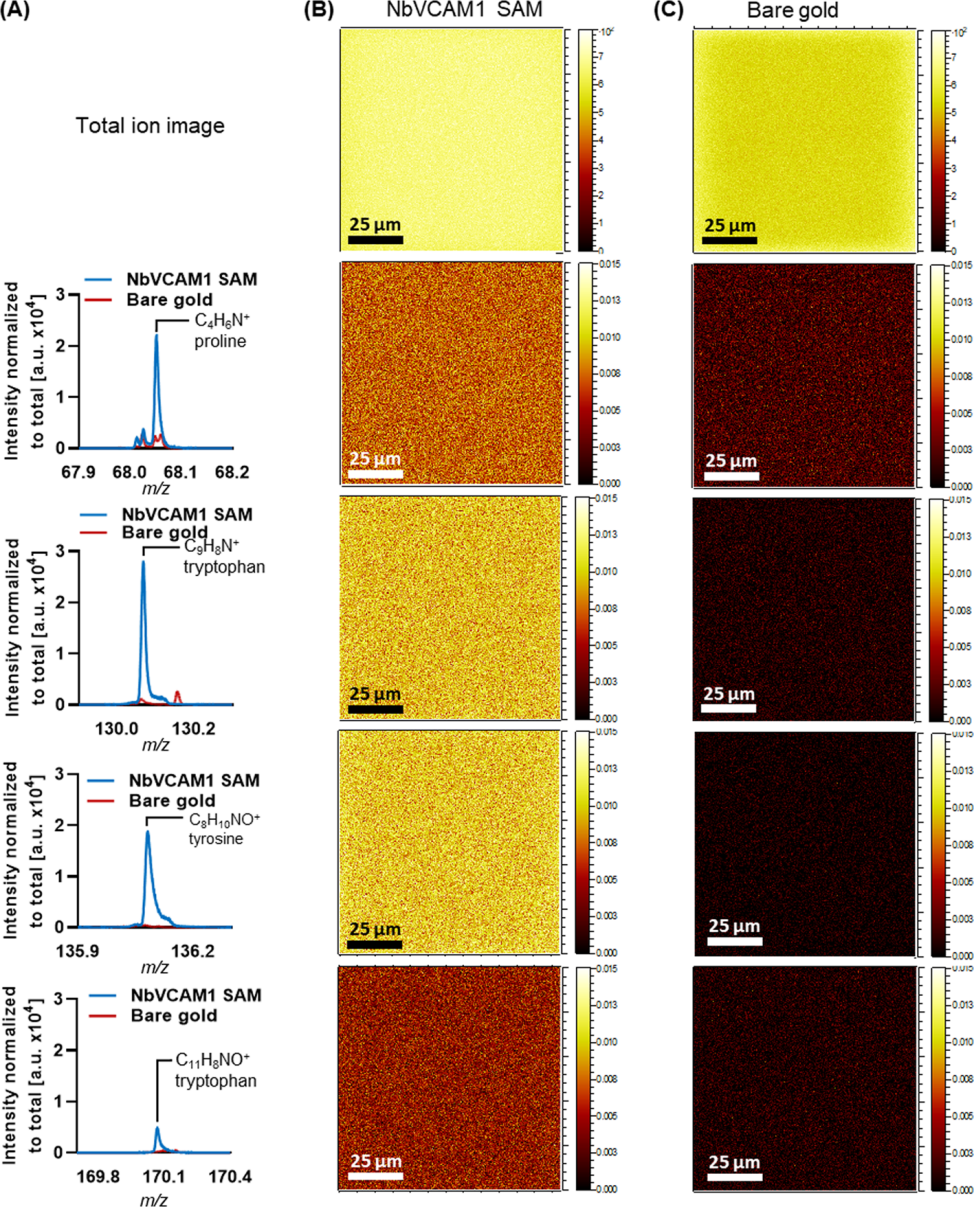
(A) Spectra overlay of amino-acid fragments (proline, tryptophan, and tyrosine) assigned in the positive polarity spectra of NbVCAM1 (blue) and gold reference (red); intensity was normalized to the total ion counts. ‘a.u’ refers to arbitrary units. (B) Respective ToF-SIMS images of gold chips incubated with NbVCAM1 SAM. (C) Amino-acid fragments are not detected in the ToF-SIMS images of bare gold reference. All the ion images have been normalized to the total ion counts.

ToF-SIMS can also directly map the distribution of different nanobody fragments on the gold surface to provide insights into the chemical interactions between the nanobody and the gold surface. Distribution maps have been plotted for amino-acid fragments (Figure 4A,B). While the lateral resolution of the technique is not sufficient to resolve individual nanobodies, the uniform ion distribution across the surface does indicate that the NbVCAM1 nanobodies are uniformly distributed on the gold surface (Figure 4B). The amino-acid fragments (proline, tryptophan, and tyrosine) were assigned according to Lhoest et al.^30^ These amino-acid fragments are not present on clean bare gold surfaces, as illustrated in Figure 4A,C.

**Figure 5:**
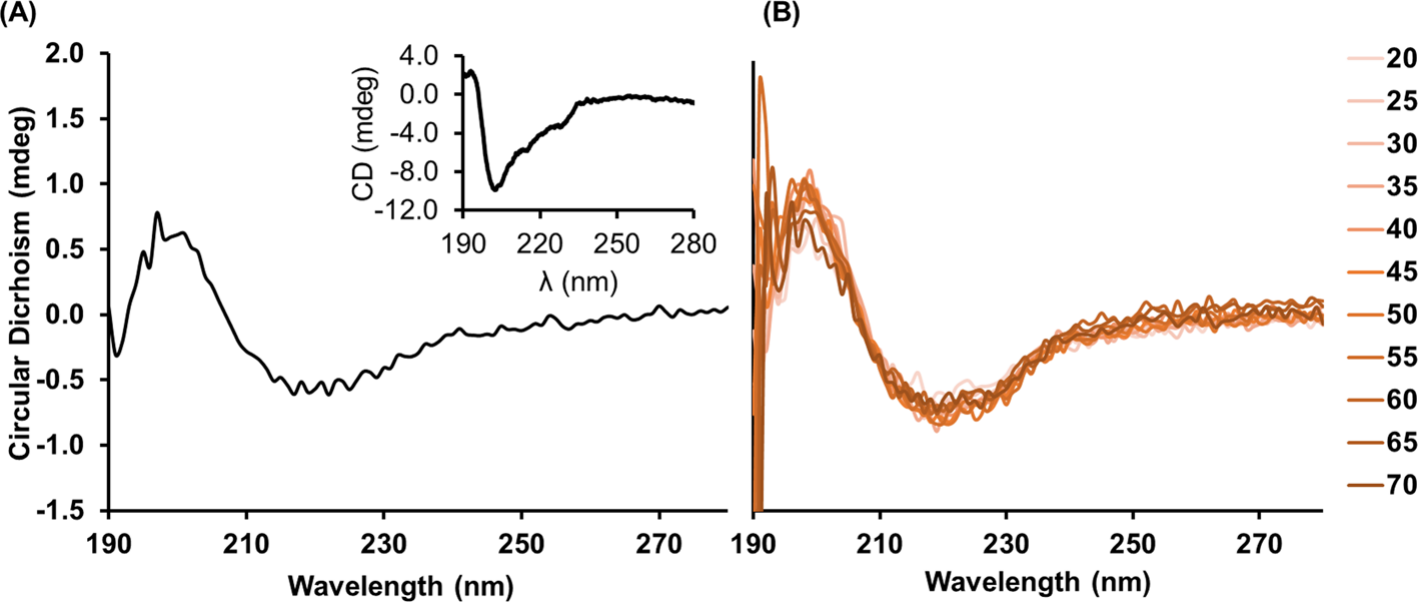
CD spectra of NbVCAM1 on a surface immobilized on a Cu^**2**+^-terminated SAM on a quartz slide. (A) Average spectra of three measurements at room temperature; inset: NbVCAM1 in solution as a control; (B) spectra overlay at temperatures ranging from 20 to 70 °C by an increment of 5 °C.

Following the analysis determining the nature of the gold-nanobody interactions, attention was turned toward understanding the structure of surface-immobilized NbVCAM1 by CD. Nanobody monolayers were formed on copper-ion-functionalized quartz slides^31^ onto which NbVCAM1 can chemisorb, in a similar manner as on gold, through the modified cysteine. Quartz was used rather than gold substrates to avoid a low signal-to-noise ratio because of the high absorption of gold in the UV region. The CD spectrum of the surface-tethered NbVCAM1, shown in Figure 5A, is similar to that of the nanobody in solution (insert in Figure 5A), both illustrating that the NbVCAM1 is composed largely of β-sheets (typified by a negative band at 217 nm and a positive band at 195 nm). These findings are in agreement with the literature^4,32,33^ that had shown that the VHH domain is composed of folded β-sheets with three loops in the regions homologous to the CDRs of the IgG VH domains. These results suggest that the nanobody conformation is not altered when they are organized in a two-dimensional monolayer. Further evidence of the conformational stability of NbVCAM1 was obtained by increasing the temperature of the NbVCAM1-functionalized quartz substrate from 20 to 70 °C (Figure 5B). The CD spectra taken at different temperatures show similar features, with the β-sheet peak remaining unchanged.

**Figure 6:**
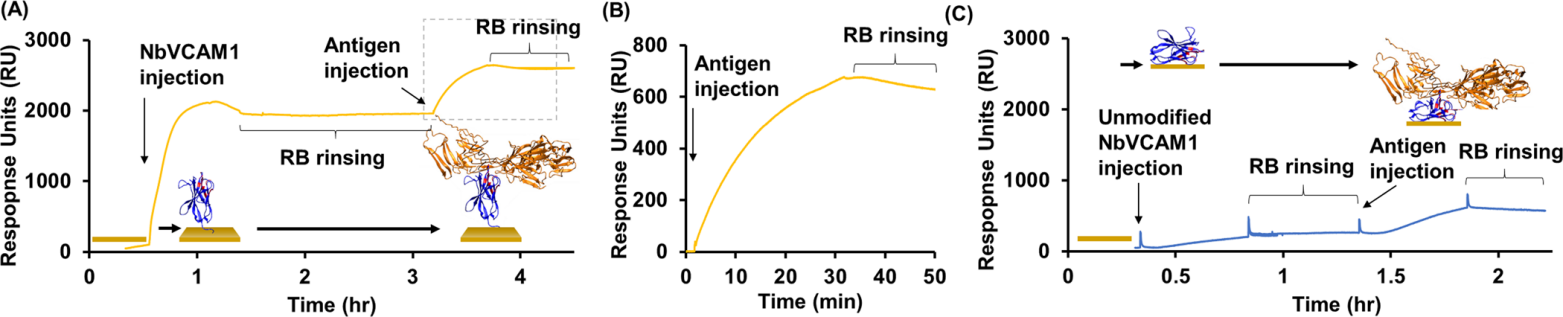
SPR results for NbVCAM1 adsorption followed by antigen injection. (A) Overview of NbVCAM1 adsorption (1 μM) followed by blank injection with running buffer (RB) and posterior antigen hVCAM1 injection (0.27 μM). (B) hVCAM1 injection response. (C) Unmodified NbVCAM1 adsorption (1 μM) followed by blank injection with RB and posterior antigen hVCAM1 injection (0.27 μM).

Having established that the nanobody retains its structure while forming a homogeneous, covalently bound monolayer on the gold surface, we assessed its antigen (hVCAM1)-binding capacity by SPR. Figure 6 portrays the formation of a NbVCAM1 monolayer in real-time, followed by hVCAM1 antigen binding. From a stable baseline of PBS flowing over a clean gold chip, an injection of NbVCAM1 shows a response of ∼2000 response units (RU), which reduces to 1800 RU as the solution is washed away with PBS. Following the initial removal of nonspecifically bound NbVCAM1, prolonged rinsing (i.e., ∼2 h) had no effect on the integrity of the NbVCAM1 monolayer, indicating the presence of a stable monolayer (Figure 6A). Because 1000 RU is equivalent to a change in the surface concentration of approximately 1 ng/mm^2^,^34^ the amount of immobilized nanobody (i.e., nanobody loading capacity on the gold surface) achieved was 1.8 ng/mm^2^, corresponding to approximately one NbVCAM1 nanobody (14.5 kDa) per 13 nm^2^. Based on the size of NbVCAM1 (i.e., 3.1 × 4.0 × 5.3 nm), the results imply the formation of a high-packed nanobody monolayer on the gold surface that enables the specific capture of the antigen. The injection of the hVCAM1 antigen produced a change in the SPR response of ∼600 RU (Figure 6B), with rinsing having a minimal effect on the final response. Following similar calculations as above, one hVCAM1 antigen (74.1 kDa) occupies an area of approximately 205 nm^2^. The hVCAM1 antigen with the dimensions of 12.9 × 7.4 × 7.6 nm is eleven times larger in volume than NbVCAM1; thus, the hVCAM1 antigen was shown to be immobilized at a high density on the NbVCAM1 monolayer. These findings confirm the high capability for the NbVCAM1 monolayer to bind its antigen (*K*_D_ = 1.61 ± 0.14 nM^5^), wherein a high degree of well-oriented nanobodies must be in place; otherwise, antigen binding would have been considerably affected. These results contrast with those obtained when an unmodified NbVCAM1 containing no cysteine at the C-terminal is immobilized on a gold surface (Figure 6C). Exposure of the gold surface to the unmodified NbVCAM1 led to a much lower SPR response of ∼180 RU, a 10-fold decrease in immobilization compared with the modified cysteine-containing NbVCAM1 at the C-terminal. These results further support the role of the added cysteine in enabling a gold-thiolate bond and the formation of a high-packed nanobody monolayer on the gold surface. Antigen binding is also reduced to half of the one observed in the NbVCAM1 monolayer. Because of the low coverage of the unmodified NbVCAM1 on the gold surface, the binding properties are difficult to interpret because the hVCAM1 antigen can be not only specifically bound to some of the nanobodies but also nonspecifically adsorbed on the gold surface.

To complement our experimental findings, we have developed a straightforward model to simulate NbVCAM1 adsorption onto a gold surface. These simulations yield insights into several aspects of nanobody adsorption, including (i) the protein’s structural behavior before, during, and after adsorption, (ii) the interactions between the protein and gold during and after adsorption, and (iii) the footprint of the adsorbed protein, which is relevant to the monolayer density that it might subsequently form. The model was built with the NbVCAM1 in water/0.15 M NaCl with a starting position at a distance of 20 Å from the gold surface. As an approach to randomize the results, the nanobody was placed at different starting orientations in a sequence of separate trajectories: with the C- to N-terminal axis approximately perpendicular to the surface (C-terminal either facing the gold surface or the bulk solution) or with the axis parallel to the surface.

**Figure 7:**
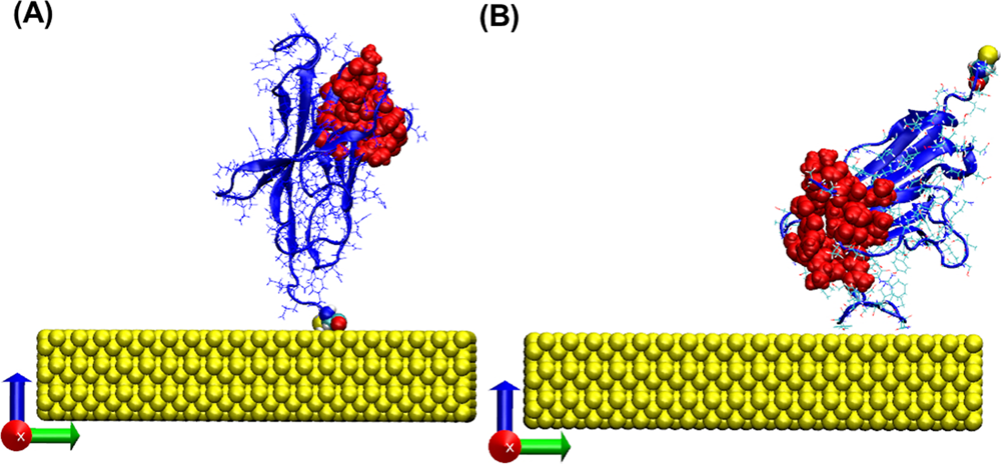
Representative VMD images from the MD simulations of the physical adsorption of NbVCAM1 on gold, resulting in (A) well-oriented and (B) nonoriented nanobodies. The NbVCAM1 representation highlights the modified cysteine at the C-terminal (white: hydrogen; cyan: carbon; red: oxygen; blue: nitrogen; and yellow: sulfur) and the amino acids that belong to the antigen binding site located at the N-terminus side in the folded domain (all atoms red) with the gold slab (yellow).

We find that the physical adsorption process can yield various adsorbed nanobody orientations, as might be expected for adsorption to a gold surface.^35^ Among these, we observe the desired orientation with the modified cysteine adsorbed to the surface and the N-terminal exposed to the solution (Figure 7A). The simulations do not directly simulate the formation of the thiolate bond but do show that these are likely to form because of the close approach of sulfur to the gold surface. Indeed, the literature widely reports that thiolate formation starts with physisorption followed by chemisorption,^36^ which stabilizes the adsorbed orientation.^37^ In contrast, the adsorbed nanobody with undesired orientations (e.g., Figure 7B) is likely to be less stable, allowing the reorientation of the nanobody over time to create a more stable thiolate-bonded structure.

**Figure 8:**
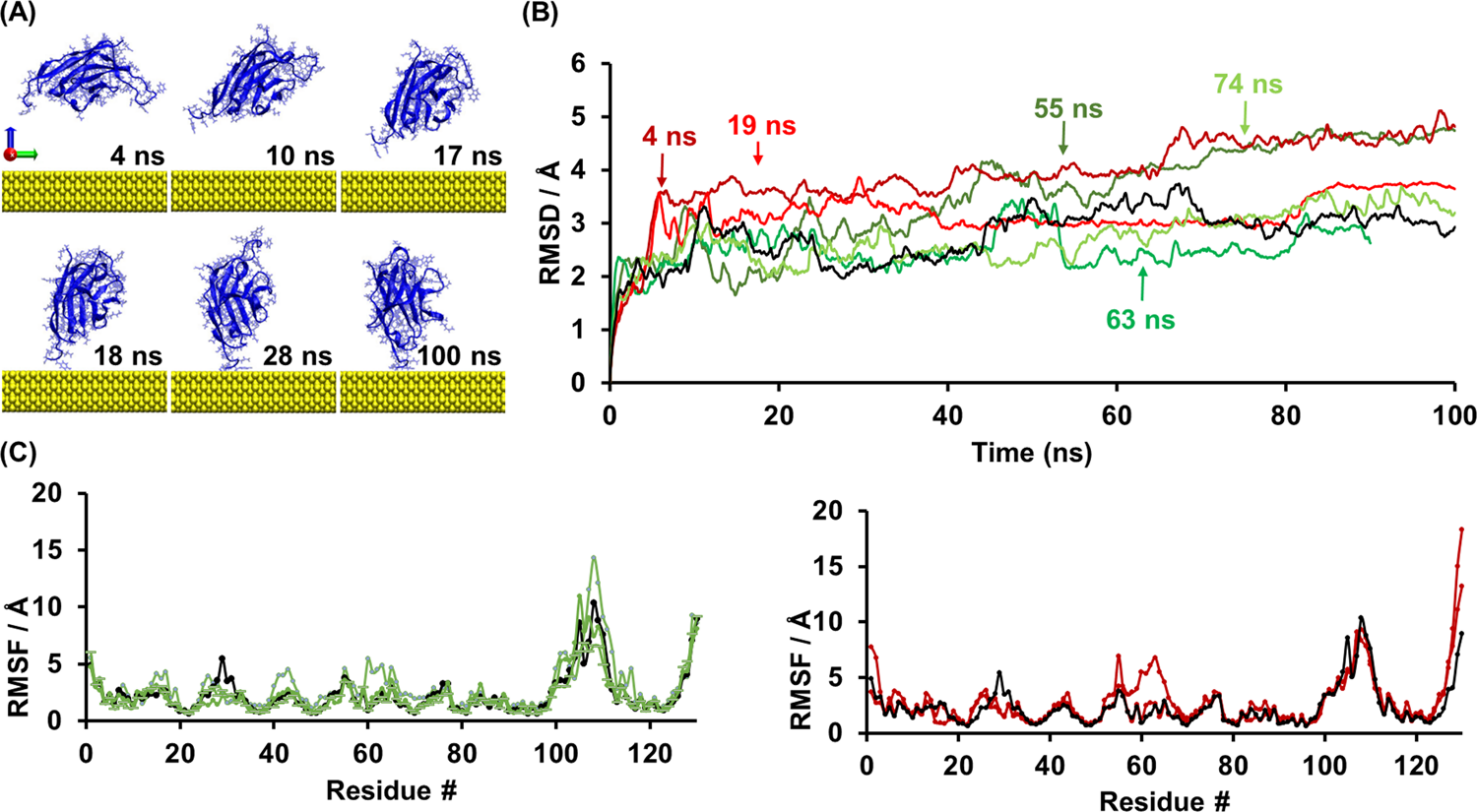
(A) MD results at various simulation time points for NbVCAM1 adsorption onto the model gold surface. (B) Root-mean-square deviation (RMSD) for the NbVCAM1 α-carbons during the adsorption process on gold over several different trajectories of 100 ns duration. The steady trend indicates that the NbVCAM1 structure was kept before, during, and after adsorption. Arrows indicate the time of adsorption in each trajectory. (C) RMS fluctuation for the same trajectories for each NbVCAM1 residue (0 to 130 residues from N to C-terminal). Residues with higher RMSF values belong to the loop areas, revealing higher mobility. (B-C) In black, the control represents the trajectory of NbVCAM1 in solution, while green and red colors represent NbVCAM1 that adsorbed nonoriented or oriented, respectively, onto the gold surface.

The root-mean-square deviation (RMSD) and root-mean-square-fluctuation (RMSF) are the tools of analysis to quantify the conformation variability within a protein.^38^ RMSD measures the degree of similarity between two 3D structures with the same number of atoms. In this case, the NbVCAM1 was compared at each step of the trajectory (step = 0.04 ns) with its initial structure (at *t* = 0). For RMSF, the RMSD is calculated for each nanobody’s residue, reflecting its fluctuations across the total trajectory. Figure 8 shows the RMSD and RMSF results for the trajectories taken before and after adsorption compared with the control trajectory obtained for NbVCAM1 in solution (with no model gold surface). At the time of adsorption (Figure 8B, identified with arrows), there is no evident spike in the RMSD data, and indeed, for the whole duration of the simulations, there is no indication of significant structural changes from the control.

Similarly, a brief analysis of the RMSF data (Figure 8C) yields the same conclusion, as the residues at the adsorption site do not vary significantly from the NbVCAM1 control. This means that the successfully adsorbed NbVCAM1 on the gold surface did not have major differences in its conformation when compared with the NbVCAM1 conformation while in solution. These findings support our experimental results showing that the nanobody structure and activity is preserved upon monolayer formation.

Thereafter, a deeper analysis of each individual residue allows us to infer which ones approach the surface and contribute to the NbVCAM1 adsorption (Figure 8C). As expected, higher RMSF values were observed for the amino acids that belong to the nanobody’s loops (especially, the CDR3 loop starting at amino-acid 99 to 111^33,39^) and at the N- and C-terminals. The exception is when one of these regions is underneath the nanobody and interacts with the gold surface, slightly reducing the flexibility and lowering the RMSF values compared to the control. On the other hand, some nonloop amino acids showed a slightly higher RMSF than the control, which meant that they contributed to the approach to the surface, resulting in an increased movement. These amino acids were SER30, 101, 104, and 126 (serine), ASN29, 106, 108, 112, 114 (asparagine), PHE53, 105 (phenylalanine), and TYR103, 115, 126, and 129 (tyrosine). LYS (lysine)^40^ and amine interactions with gold^41^ have been previously reported, and likewise, with intermittent contact, LYS43, 44, and 76 contributed to the NbVCAM1 immobilization at the surface.

**Figure 9:**
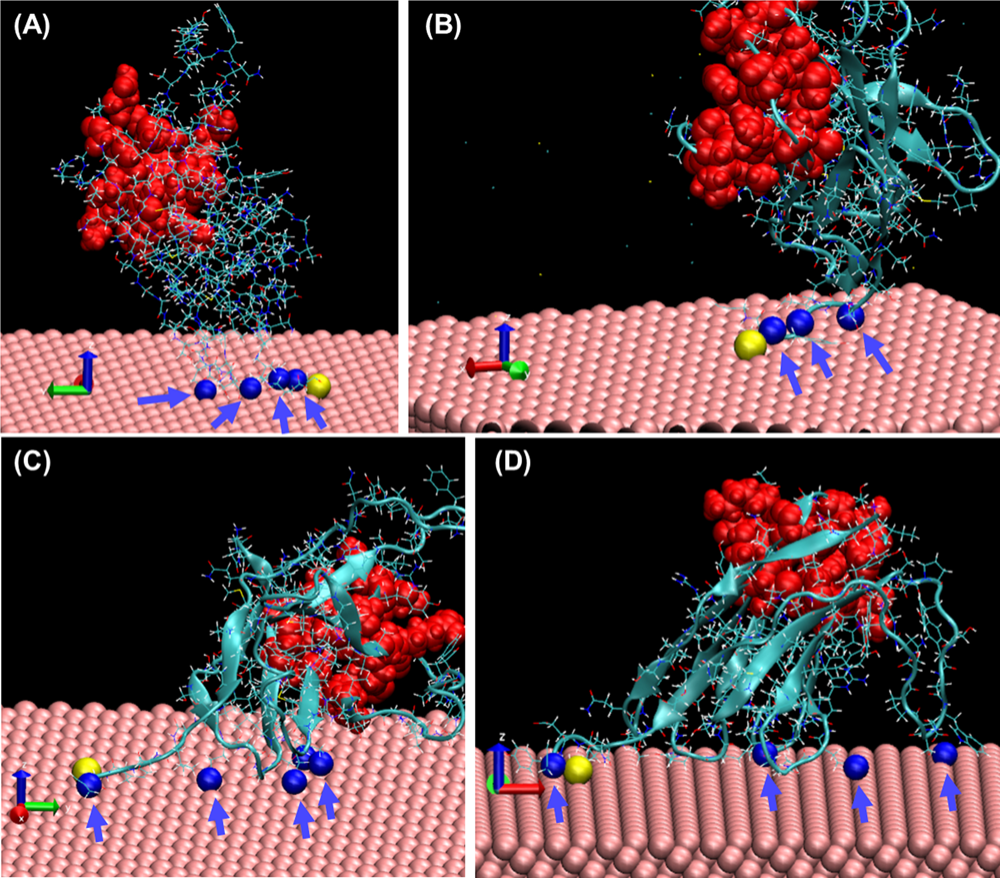
Representative VMD images of NbVCAM1 immobilized by a thiolate bond (A, B) and physically adsorbed by the modified cysteine (C, D). Highlighted are the NbVCAM1’s nitrogen–gold interactions, the sulfur at the modified cysteine (yellow), and the binding site (red). Arrows point to the closest nitrogen atoms (blue) to the gold surface.

Finally, in order to understand how the adsorbed NbVCAM1 orientation might change over time and how it might be affected by the creation of the thiolate bond, a second model was developed with NbVCAM1 tethered by a thiolate bond to the gold surface (Figure 9A,B). This model allowed us to observe the flexing of the nanobody above the surface. The simulations reveal nitrogen–gold interactions from the amino acids (CYS130, LEU127, TYR129 and GLN13) near to the thiolate bond site, which are involved in creating a rather tilted orientation. We note that these features are also present in the simulations where the thiolate bond was not created, with additional nitrogen interactions (LYS44, 45, GLN87, ASN84, 85, 87, 108) (Figure 9C,D). These observations further confirm our earlier findings from the SIMS analysis, wherein nanobody immobilization on the gold surface can be attributed to the simultaneous formation of a gold–thiolate bond and nitrogen–gold interactions.

## Conclusions

The nanobodies’ relatively small size (∼15 kDa) and their prominent stability meet the highly desired characteristics when designing and developing a vast range of biosensors and diagnostic tools. In order to take advantage of these characteristics, we hypothesized that adding a modified cysteine would promote direct surface functionalization on gold. Our findings demonstrated, through ToF-SIMS and 3D OrbiSIMS, the formation of a thiolate bond between the NbVCAM1 nanobody and the gold surface. The secondary ion imaging results also provided compelling evidence of the formation of a homogenous, stable, and well-packed nanobody monolayer. Our experimental and theoretical findings furthermore support the presence of a high degree of well-oriented nanobodies on the gold surface, leading to a high capacity for antigen binding. The strategy for nanobody immobilization is simple and effective and can be adopted to other highly relevant nanobody–antigen systems. Considering all these attributes, this work opens up new avenues for the design and scalable fabrication of stable, reliable, and robust biosensing platforms for a wide range of medical, biotechnological, environmental, and food applications.

## Supporting Information Available

Experimental procedures for nanobody monolayer formation and characterization, and a description of the MD simulations methodology (PDF)
