# Supplementary material for: Direct Immobilization of Engineered Nanobodies on Gold Sensors
Source: ACS Appl Mater Interfaces. 2021 Apr 12;13(15):17353–60. doi: 10.1021/acsami.1c02280 (PMC8153533; doi:10.1021/acsami.1c02280)
Supplement: Supplementary file 1 — am1c02280_si_001.pdf [file am1c02280_si_001.pdf]

SUPPORTING INFORMATION

Direct immobilization of engineered nanobodies on gold sensors

Bárbara Simões,[†] Wanda J. Guedens,[§] Charlie Keene,[†] Karina Kubiak-Ossowska,[#] Paul Mulheran,[¥] Anna M. Kotowska,[‡] David J. Scurr,[‡] Morgan Alexander, [‡] Alexis Broisat,[‖] Steven Johnson,[±] Serge Muyldermans,[₸] Nick Devoogdt,[⅄] Peter Adriaensens,[§] Paula M. Mendes[†,*]

[†]School of Chemical Engineering, University of Birmingham, Edgbaston, Birmingham, B15 2TT, UK.
[§]Institute for Materials Research (IMO), Hasselt University, BE-3590 Diepenbeek, Belgium.
[#]Department of Physics and [¥] Department of Chemical & Process Engineering, University of Strathclyde, Glasgow, G1 1XQ, UK.
[‡]School of Pharmacy, University of Nottingham, Nottingham, NG7 2RD, UK.
[±]Department of Electronic Engineering, University of York, York, YO105DD, UK.
[‖]Laboratory of Bioclinical Radiopharmaceutics, Université Grenoble Alpes, Inserm, CHU Grenoble Alpes, LRB, 38000 Grenoble, France.
[₸]Cellular and Molecular Immunology laboratory, Vrije Universiteit Brussel (VUB), BE-1090 Brussels, Belgium.
[⅄] In vivo Cellular and Molecular Imaging laboratory, Vrije Universiteit Brussel (VUB), BE-1090 Brussels, Belgium.

*Professor Paula M Mendes, School of Chemical Engineering, University of Birmingham, Edgbaston, Birmingham, B15 2TT, UK, Email: p.m.mendes@bham.ac.uk.

Materials and Methods

Chemicals

All the chemicals were bought from Sigma Aldrich with exception of hVCAM1 antigen, which was purchased from PeproTech.

Preparation of NbVCAM1 nanobodies

Nanobody cAbVCAM1-5, here further denoted as NbVCAM1, was previously isolated from an immune dromedary library as a lead compound for nuclear imaging of VCAM1-expressing atherosclerotic plaques in mice and in patients.[1] The NbVCAM1 nanobody was extended with amino acids LEY at the C-terminus, expressed as a chimeric protein (fusion with an intein and a chitin binding domain) in the *E. coli* SHuffle® T7 strain and was subjected to EPL-mediated cleavage with the cysteine-alkyne linker to produce the C-terminally alkynated NbVCAM1-LEY, i.e., NbVCAM1-LEYC-alkyne, as previously described[2] and referred in this paper as simply NbVCAM1. The structure of the cysteine-alkyne linker was characterized by NMR spectroscopy.

Gold substrates

Gold sensor chips used in the SPR experiments were acquired from Reichert Technologies - Ametek Inc (USA) and consisted of polycrystalline gold surfaces (50 nm) on glass substrates sized 1 cm x 1 cm. For ellipsometry, contact angle and ToF-SIMS studies, gold (100 nm thickness and rms roughness < 2,5 nm) on silicon <100> wafers pre-coated with titanium were acquired from George Albert PVD (Germany) and cut into 1 cm x 1 cm pieces using a diamond scriber.

Substrate treatment

Before functionalization, the gold substrates were submerged for 10 min in a strong oxidizing piranha solution (70% H_2SO_4, 30% H_2O_2) to remove organic residues (*Caution: Piranha solution reacts violently with all organic compounds and should be handled with care*). Subsequently, the gold substrates were rinsed with copious amount of Ultra High Pure (UHP) water and HPLC ethanol and lastly dried under argon.

NbVCAM1 self-assembly monolayers (SAMs)

The lyophilized NbVCAM1 was kept at -20°C until further use. Freshly piranha cleaned gold chips were incubated with a solution of 1 µM NbVCAM1 diluted in 1x PBS for 24 h at room temperature on a moving plate. As controls, gold chips were immersed in either 1x PBS or HPLC ethanol solutions under the same conditions.

Contact angle

The advancing and receding contact angles were obtained with the instrument OneAttension using the sessile drop analysis mode. The drop volume reached 4-10 µL at a rate of 0.5 µL/sec. Two to three measurements per chip (duplicates) were performed in different chip locations.

Ellipsometry

Ellipsometry measurements were obtained with a J.A. Woollam alpha-SE instrument using gold on silicon wafers. Data analysis used the Cauchy model that considers three layers: Ambient/Monolayer/Substrate. The refractive index was fixed at 1.5. Each chip was measured before and after functionalization. Data was fitted with the software CompleteEASE, with a defined resolution of 0.1 eV. Four measurements per chip (duplicates) were performed in different chip locations.

Time-of-flight-secondary ion mass spectroscopy (ToF-SIMS) and three dimensional Orbitrap secondary ion mass spectroscopy (3D OrbiSIMS)

ToF-SIMS spectra were acquired using a ToF IV (IONTOF GmbH) instrument with 25 keV Bi_3^+ primary ion beam raster over 500 × 500 µm area. Additional high lateral resolution ToF-SIMS imaging was acquired using 3D OrbiSIMS instrument with 25 keV Bi_3^+ primary ion beam and delayed extraction. Two 256 × 256 pixel images over area of 100 × 100 µm were acquired on two replicates of each sample type. Measurements were performed in both positive and negative mode. Positive mode spectra were calibrated to: CH_3^+, $C_7H_7^+$, Au_3^+. Negative mode spectra were calibrated to: CH^-, CN^-, CNO^-, Au_3^-. Two measurements were taken for each sample and each polarity.

3D OrbiSIMS measurements were acquired using 20 keV Ar_{3000}^{+} as primary ion beam. The current of the primary beam was 220 pA. Each spectrum was acquired from an area of 300 × 300 µm using random raster mode and the crater size was 381.9 × 381.9 µm. The spectra were collected in positive and negative mode, in mass ranges 50-750 *m/z* and 150-2250 *m/z*. Target potential was set to +57.5 V for positive mode and -57.5 V in negative mode. Two separate areas were analyzed on each sample and two replicates of each sample type were analyzed. Each measurement lasted 30 scans, the total ion dose per measurement was 1.6 × 10^{10}. Mass resolving power was set to 240,000 at 200 *m/z*.

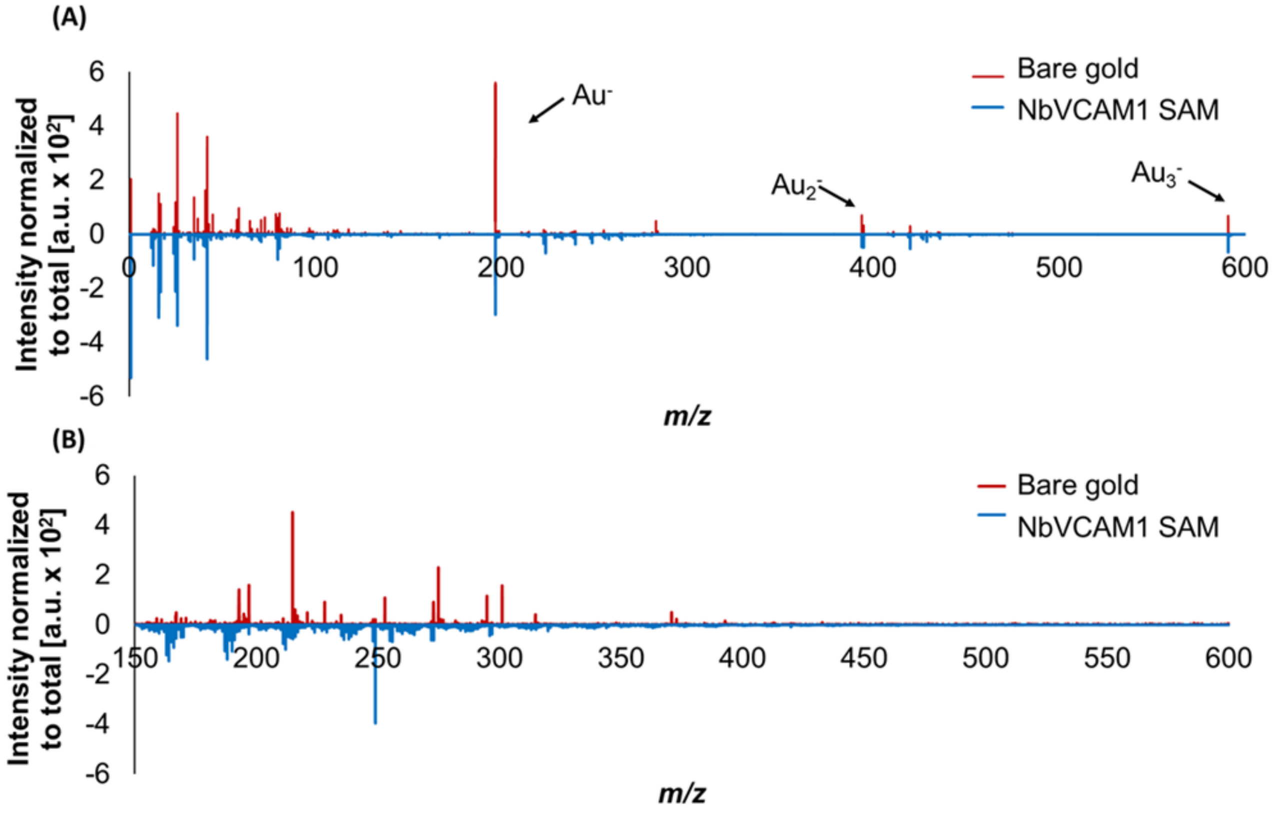

Figure S1 - (A) ToF-SIMS and (B) 3D OrbiSIMS survey spectra for the NbVCAM1 SAM (blue) and gold reference (red).

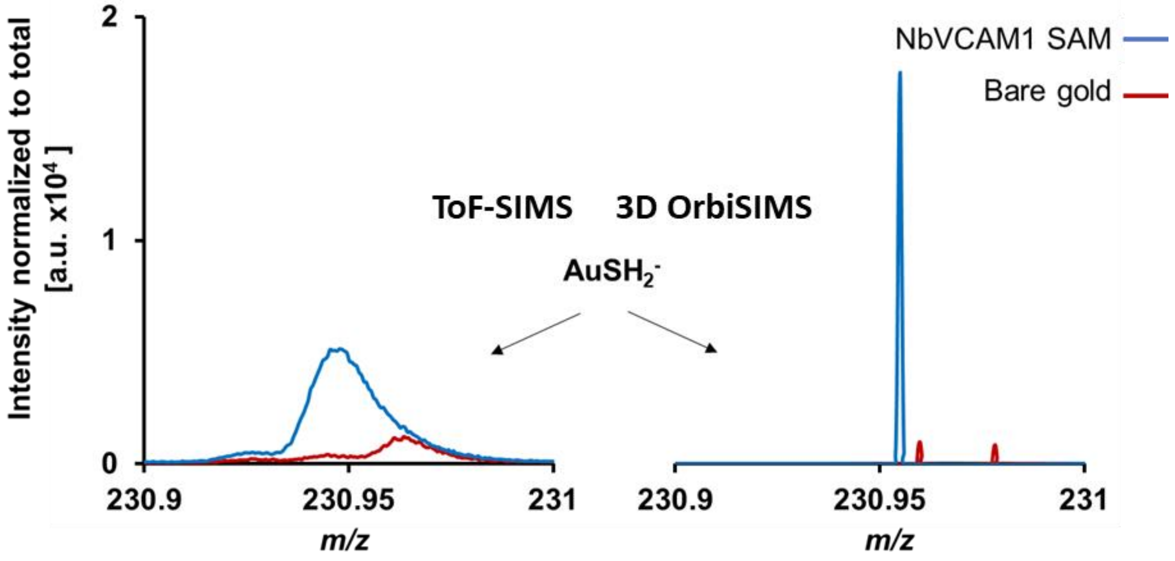

Figure S2 - Representative spectra for methods comparison between ToF-SIMS and 3D OrbiSIMS. The mass resolving power of the 3D OrbiSIMS allows to assign peaks representing the AuS^- bond with higher confidence than in the ToF-SIMS spectra.

Circular dichroism (CD)

The circular dichroism was performed on a NbVCAM1 SAM surface and with NbVCAM1 in solution, allowing confirmation of its conformation. Studies of the NbVCAM1 in 1xPBS were performed in a Jasco J-1500, using a nanobody concentration of 1 mg/ml, at room temperature. CD analysis of the NbVCAM1 SAM surface were performed using a Chirascan plus. Piranha cleaned quartz slides were incubated overnight with a 4% solution of mercapto-trimethoxysilane (MPTES) in IPA, allowing thiol functionalization. Following rinsing with IPA to remove the excess of MPTES, the slides were immersed in 15 mM copper perchlorate solution in H_2O for 15 minutes to provide a Cu^{1+} ion surface. Finally, the slides were incubated with NbVCAM1 0.1 mg/ml in 10 mM phosphate buffer, pH 7.9 for 1 h, and rinsed with the same buffer. Three slides were loaded into a quartz cuvette. All measurements were performed with a 10 mM phosphate buffer pH 7.9.

Molecular dynamic (MD) simulations

Simulation model for nanobody adsorption

In order to study and analyze the adsorption of NbVCAM1 on a model gold surface, the NAMD2.12[3] software was used with periodic boundary conditions, the TIP3P water model, the CHARMM27 force-field with a 12 Å cut-off for short-range potentials, and smooth particle mesh Ewald summation for the electrostatics. Visual molecular dynamics (VMD) software version 1.9.1 was employed to analyze the results.[4] The NbVCAM1 structure was obtained from Phyre[2] (protein homology/analogy recognition engine) software,[5] that predicted the structure according to the amino-acid sequence.

The predicted structure considered 127 residues (amino-acids), not including the C-terminal termination –LEY linker and cysteine-alkyne linker, which were both added with NAMD2.12 software. This is due to the linker -LEY being an addition to the natural sequence, added artificially, as well as the cysteine-alkyne- linker (EPL).[6] The predicted structure has a total net charge of +2e and a disulfide bridge which was kept throughout the simulations. The residues belonging to the binding site were identified at the N-terminal by the open source platform for ligand detection *Fpocket* [7].

The simulations were performed in a NaCl solution and with a slab of the gold (81 Å x 86 Å x 14 Å) that consisted of Au atoms. The close packed gold surface of Au,[8] as already reported elsewhere,[9] has been created as a face-centered cubic crystal (fcc) with lattice parameter 4.078 Å. CHARM-METAL[10,11] has been used for gold force-field parameters. The gold atoms were kept immobile during the simulation.

The simulations start with the protein above the solid surface, with a minimum protein-gold separation of 20 Å, so that the protein is free to diffuse before it contacts the neutral gold surface. In order to not bias the adsorption process, the protein starts in different orientations in different trajectories, as illustrated in Figure S3. In P1, the N-C axis is normal to the surface with the C-terminal facing away from the surface, and in P2 it faces towards the surface. In P3, the N-C axis lies parallel to the surface. In all these starting configurations, the simulation box is then solvated with the TIP3P waters, neutralized by addition of one Cl^- ion, and then brought to 150 mM NaCl concentration.

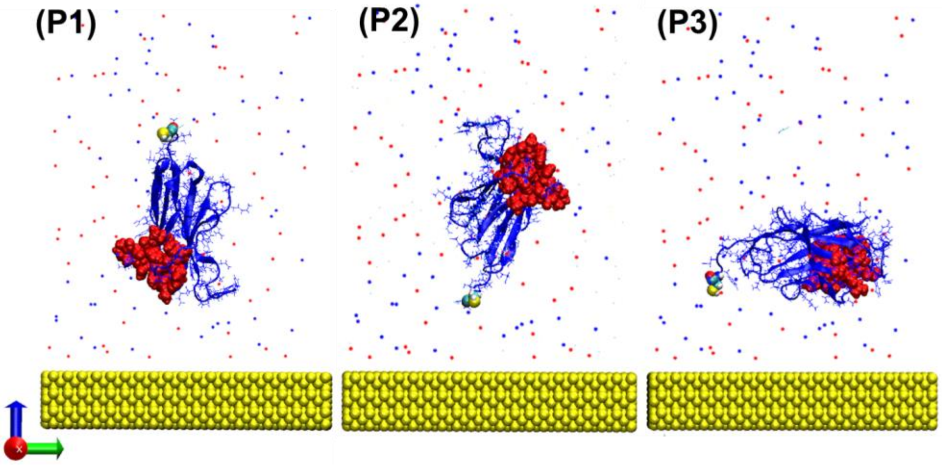

Figure S3 - Starting positions for MD simulations of NbVCAM1 adsorption at the surface. The gold atoms (yellow), the binding site (red) and the modified cysteine at the C-terminus (oxygen – red; carbon – cyan; nitrogen – blue; hydrogen – white and sulfur - yellow) are represented as van der Walls (VDW) spheres, while the other elements of the protein are represented by lines and new cartoon superposed. The NaCl ions in solution are also shown, but the water is not for clarity.

The C-terminal modified cysteine is important due to the possibility for a thiolate bond forming with the surface, anchoring the nanobody in a favorable orientation for antigen binding. While these classical MD simulations do

not attempt to create the thiolate bond during the simulation, it is still important to understand whether its formation is favourable due to the initial physical adsorption of the nanobody to the surface. At least three trajectories from each initial position (P1, P2, P3) were performed. The system minimization was performed sequentially in two steps, first allowing water and ion movements, and then freeing the protein. The system is then heated to 310 K temperature over 30 ps, followed by 270 ps equilibration at constant temperature. Finally, the production simulations were performed for 100 ns with a time-step of 2 fs. Periodic Boundary conditions and NVT ensemble were applied in the simulations together with the smooth Particle Mesh Ewald (SPME)[12] for the Coulomb interactions. For ionizable residues the most probable charge states at pH 7 were chosen and no additional restrictions on momentum were applied.

Simulation model for nanobody anchored by a thiolate bond to gold

So far it is not possible to simulate a bond formation event such as the thiolate bond between the nanobody and gold surface, hence this bond had to be created with the force field parameters described below (Table S1), which were added to the parameters and topology files.

Table S1 - Force field parameters.

**Bond stretching**				REF
**Bond**	b_0(Å)	$K_b(kcal/(mol/Å^2))$		
S-Au	2.4	1000		13
**Angle bending**				
**Angle**	θ_0(°)	$k_\theta(kcal/(mol/rad^2))$		
Au-S-C	120.0	124.28		ChemBio 3D; 13
**Torsion**				
**Dihedrals**	K_ϕ $(kcal/mol)$	n	δ (°)	
Au-S-C-C	0.310	3	0.00	Like S-S-C-C
Au-S-C-C	0.310	3	0.00	Like S-S-C-C
Au-S-C-H	0.158	3	0.00	Like S-S-C-H

Analysis: RMSD, RMSF

Both root-mean-square distance (RMSD) and fluctuations (RMSF) are tools of analysis to quantify the variability on conformation within a protein.[14] RMSD measures the degree of similarity between two protein three-dimensional (3D) structures with the same number of atoms. It is defined as

$$RMSD = \sqrt{\frac{\sum_{i=1}^{Natoms} |\vec{r}_i(t_1) - \vec{r}_i(t_2)|^2}{Natoms}}$$

Where the parameter $Natoms$ is the number of atoms in the protein structure and $\vec{r}_i(t_1)$ is the position of the ith atom at a given time t. The RMSD calculation treats two protein structures to be compared as two rigid bodies (no internal flexibility allowed), then overlaps (aligns) using translations and rotations. In this case, the nanobody

NbVCAM1 is compared with itself, between the final and initial defined structures. Herein, the RMSD results were applied to the alpha carbons that composed every residue of the nanobody or oligopeptide.

For RMSF, the RMSD is calculated for each nanobody's residue. It reflects each residue's mobility during the MD trajectory, by reporting an amplitude of residue movement (fluctuation) from the average position (in the aligned structures) over the total length of the MD trajectory. The time average for the atoms belonging to the same residue were calculated from the formula

$$RMSF_k = \sqrt{\langle \frac{\sum_{i=1}^{N_k} |\vec{r}_i(t) - \langle \vec{r}_i \rangle_T|^2}{N_k} \rangle_T}$$

Where $\vec{r}_i(t)$ is the position of the atom i in residue k at the time t, N_k is the number of atoms in the residue, and $\langle \vec{r}_i \rangle_T$ is the time average over the trajectory.

Surface plasmon resonance (SPR)

SPR experiments allowed monitoring in real-time the NbVCAM1 immobilization onto the surface, followed by antigen binding. Experiments were performed in a semi-automatic Reichert Technologies SPR at the set temperature of 25 ºC. Initial traces were stabilized before each experiment at a flow rate of 100 µl/min with the running buffer (RB, 1xPBS). Blank injections (with RB) were 10 to 15 min long and were performed before each experiment to remove any impurities and after each experiment to stabilise the final response. Each injection started with a burst flow of 1500 µl/min for 10 sec of hVCAM1 (0.27 µM), followed by another injection at a flow rate of 8 µl/min for 30 min. The rising step started by injecting the RB at a flow rate of 8 µl/min for 20 min and then changing it to 100 µl/min.

References

1. Broisat, A.; Hernot, S.; Toczek, J.; De Vos, J.; Riou, L. M.; Martin, S.; Ahmadi, M.; Thielens, N.; Wernery, U.; Caveliers, V.; Muyldermans, S.; Lahoutte, T.; Fagret, D.; Ghezzi, C.; Devoogdt, N., Nanobodies Targeting Mouse/Human Vcam1 for the Nuclear Imaging of Atherosclerotic Lesions. *Circ. Res.* **2012,** *110* (7), 927-937.
2. Ta, D. T.; Redeker, E. S.; Billen, B.; Reekmans, G.; Sikulu, J.; Noben, J. P.; Guedens, W.; Adriaensens, P., An Efficient Protocol Towards Site-Specifically Clickable Nanobodies in High Yield: Cytoplasmic Expression in Escherichia Coli Combined with Intein-Mediated Protein Ligation. *Protein Eng. Des. Sel.* **2015,** *28* (10), 351-63.
3. Phillips, J. C.; Braun, R.; Wang, W.; Gumbart, J.; Tajkhorshid, E.; Villa, E.; Chipot, C.; Skeel, R. D.; Kalé, L.; Schulten, K., Scalable Molecular Dynamics with Namd. *J. Comput. Chem.* **2005,** *26* (16), 1781-1802.
4. Humphrey, W.; Dalke, A.; Schulten, K., Vmd: Visual Molecular Dynamics. *J. Mol. Graph.* **1996,** *14* (1), 33-38.
5. Kelley, L. A.; Mezulis, S.; Yates, C. M.; Wass, M. N.; Sternberg, M. J. E., The Phyre2 Web Portal for Protein Modeling, Prediction and Analysis. *Nat. Protoc.* **2015,** *10* (6), 845-858.

6. Ta, D. T.; Redeker, E. S.; Billen, B.; Reekmans, G.; Sikulu, J.; Noben, J. P.; Guedens, W.; Adriaensens, P., An Efficient Protocol Towards Site-Specifically Clickable Nanobodies in High Yield: Cytoplasmic Expression in Escherichia Coli Combined with Intein-Mediated Protein Ligation. **2015,** *28* (10), 351-363.
7. Le Guilloux, V.; Schmidtke, P.; Tuffery, P., Fpocket: An Open Source Platform for Ligand Pocket Detection. *BMC Bioinform.* **2009,** *10* (1), 168.
8. Cooper, E.; Krebs, F.; Smith, M.; Raval, R., The Interaction of Amino Acids with Metals: Methionine on Gold. *J. Electron. Spectros. Relat. Phenomena* **1993,** *64-65*, 469-475.
9. Kubiak-Ossowska, K.; Mulheran, P. A.; Nowak, W., Fibronectin Module Fn(Iii)9 Adsorption at Contrasting Solid Model Surfaces Studied by Atomistic Molecular Dynamics. *J. Phys. Chem. B* **2014,** *118* (33), 9900-8.
10. Heinz, H.; Farmer, B. L.; Pandey, R. B.; Slocik, J. M.; Patnaik, S. S.; Pachter, R.; Naik, R. R., Nature of Molecular Interactions of Peptides with Gold, Palladium, and Pd-Au Bimetal Surfaces in Aqueous Solution. *J. Am. Chem. Soc.* **2009,** *131* (28), 9704-14.
11. Feng, J.; Pandey, R. B.; Berry, R. J.; Farmer, B. L.; Naik, R. R.; Heinz, H., Adsorption Mechanism of Single Amino Acid and Surfactant Molecules to Au {111} Surfaces in Aqueous Solution: Design Rules for Metal-Binding Molecules. *Soft Matter* **2011,** *7* (5), 2113-2120.
12. Kastenholz, M. A.; Hünenberger, P. H., Influence of Artificial Periodicity and Ionic Strength in Molecular Dynamics Simulations of Charged Biomolecules Employing Lattice-Sum Methods. *J. Phys. Chem. B* **2004,** *108* (2), 774-788.
13. Rai, B.; Sathish, P.; Malhotra, C. P.; Pradip; Ayappa, K. G., Molecular Dynamic Simulations of Self-Assembled Alkylthiolate Monolayers on an Au(111) Surface. *Langmuir* **2004,** *20* (8), 3138-3144.
14. Al Qaraghuli, M. M.; Kubiak-Ossowska, K.; Mulheran, P. A., Thinking Outside the Laboratory: Analyses of Antibody Structure and Dynamics within Different Solvent Environments in Molecular Dynamics (Md) Simulations. *Antibodies* **2018,** *7* (3), 21.
